# Early career experiences of international medical program graduates: An international, longitudinal, mixed-methods study

**DOI:** 10.1007/s40037-022-00721-z

**Published:** 2022-07-26

**Authors:** Emmaline E. Brouwer, Tiuri R. van Rossum, Janneke M. Frambach, Erik W. Driessen

**Affiliations:** Department of Educational Development and Research, School of Health Professions Education (SHE), Maastricht University, Maastricht, The Netherlands

**Keywords:** Internationalization, International medical programs, Curriculum evaluation, Transitions

## Abstract

**Introduction:**

Increasingly medical students pursue medical education abroad. Graduates from International Medical Programs (IMPs) practice globally, yet how to prepare students for an unknown international environment is complex. Following IMP graduates throughout their early careers, this study offers insights into gaps in current undergraduate education.

**Methods:**

In this international, longitudinal, mixed-methods study, 188 graduates from seven IMPs completed baseline surveys on career choice and job preparedness. Forty-two participants completed follow-up until three years after graduation. Nine graduates participated in semi-structured interviews on individual experiences and the evolution of their perspectives. The multiphase, sequential design allowed data collected at baseline to inform further data collection instruments.

**Results:**

Two typical student profiles emerged. The first depicts a student who, despite the challenges of studying abroad, pursues a medical degree ‘anyhow’, with a common aim of practicing in their home country. The other deliberately selects an IMP while envisaging an international career. Two years after graduation, the majority (> 70%) of our participants were practicing in a country other than their country of training. They reported challenges around licensing, the job application process and health system familiarization. Participants’ experiences point towards potential curriculum adaptations to facilitate cross-border transitions, including career guidance, networking and entrance exam preparation.

**Discussion:**

IMP graduates lack support in practical aspects of career orientation and international exposure. Most IMPs essentially prepare their graduates for a career elsewhere. Gaps and challenges that IMP graduates experience in this cross-border career transition entail a responsibility for preparation and guidance that is currently lacking in IMP curricula.

**Supplementary Information:**

The online version of this article (10.1007/s40037-022-00721-z) contains supplementary material, which is available to authorized users.

## Introduction

In the past decade, increasing numbers of students have pursued medical education outside their home country [1]. European, Asian and Caribbean universities may offer an attractive alternative for students to numerus clausus policies and high tuition fees at home [1, 2]. Over two hundred so-called International Medical Programs (IMPs) in these regions offer English language medical education, frequently through translated versions of pre-existing curricula in the local language [3–5].

IMPs are a response to increasing global standardization of medical school accreditation [6, 7] and regional agreements on recognition of qualifications [1, 8], allowing IMP graduates to end up in many different locations of practice. Exact numbers are unknown, but case studies suggest that most graduates leave the country where they studied upon completing the IMP [1].

This transition, whether to a graduate’s home country or onwards to a third country, is not without challenges. Scholarly work on International Medical Graduates (IMGs, physicians working in a country other than their country of training), highlights the adaptation difficulties these graduates face when entering a new healthcare context, especially around language, intercultural communication and health system familiarization [9–11]. The IMG literature focuses on experiences in the post-graduate work context. Yet, it is essential to also consider the undergraduate context that precedes cross-border transitions, and to explore how these programs can best prepare their candidates for future international work.

Against this background, the challenges of curriculum design for IMPs become apparent. It is questionable whether a merely translated local curriculum best meets the specific needs of the diverse student group in IMPs [12]. Yet, identifying training needs and learning objectives for this particular student population is highly challenging. Insights from research on curriculum internationalization [13], global health competency frameworks [14, 15] and global citizenship education [16, 17] provide relevant suggestions for curriculum adaptations like intercultural communication and healthcare system comparison to prepare students for a globalized world. Yet, how such adaptations relate to graduate experiences is unknown.

The IMP context thus offers a unique opportunity to fill the gap between earlier research in the post-graduate IMG context and undergraduate curriculum internationalization. Studying the experiences of IMP graduates can give valuable insights in career preparedness and highlight gaps in their current undergraduate education. Previous research has shown that following these experiences longitudinally through their early career gives the most reliable information on career choice and curriculum evaluation [18, 19]. Better insight into graduates’ perceived obstacles and early career experiences will inform curriculum design decisions to help prepare students best for the transition to international clinical practice.

This longitudinal mixed-method study aimed to explore the alignment of IMPs to early career requirements as experienced by recent IMP graduates. The research questions were: (1) What are the career choices (location, specialty) made by IMP graduates and what are the motivations behind these choices? (2) What (international) job requirements do they encounter? (3) How do they evaluate the IMP curriculum’s success in preparing them for the job market?

## Methods

### Design

We conducted a longitudinal mixed-methods study, employing surveys and semi-structured interviews at four data collection moments spanning 2017–2020. The study takes a pragmatic approach to provide a more complete understanding of the research problem from multiple perspectives [20]. The longitudinal design helped us observe and understand changes in career preference and curriculum evaluation over time. This study was first approved by the Ethical Review Board of the Netherlands Association for Medical Education (ref. no. 00837). We then sought and obtained further ethical approval at each participating institution where required, according to local regulations.

### Setting

Seven International Medical Programs in Europe and Asia agreed to invite their 2017 and 2018 graduates for this study (see Tab. 1). We defined an IMP as a program (1) that actively recruits international students and (2) that offers a separate program in parallel to a ‘regular’ local curriculum. The main teaching activities in these programs occur in English, as opposed to the regular program in the national language.

**Table 1:** Overview and characteristics of participating universities

University	Country	Start date of international program	Cohort size	Private or Public	Curriculum characteristics
Gadjah Mada University	Indonesia	2002	80	Public	5Y / I / PBL
Jagiellonian University	Poland	1994	60	Private	4Y / D / L
Maastricht University	The Netherlands	2009	60	Both	6Y / I / PBL
Riga Stradins University	Latvia	2010	200	Public	6Y / D / L
University of Pavia	Italy	2009	100	Public	6Y / D / L
University of Pécs	Hungary	1985	150	Private	6Y / D / L
Zhejiang University	China	2004	80	Public	6Y / D / L

*Y* duration in years, *D* discipline based, *I* integrated, *L* lecture based, *PBL* problem based learning

### Sampling and recruitment

We employed a three-step sampling procedure, aimed at maximum variation in institutional characteristics and participating graduates’ background. First, we recruited the institutions. In a previous study, we assembled a list of over 200 medical programs that fit the IMP definition and compiled a subset of 50 that offered extensive information online giving insight in program duration, teaching format, size, public/private, age, and admission criteria [5]. Of these 50 institutions, we selected 20 that varied with regard to these elements and emailed their alumni offices or education research departments. Ten did not reply, three declined participation, and seven institutions agreed to participate.

Second, we invited survey participants. In five of the seven institutions, a local coordinator emailed all graduating IMP students in 2017 and 2018. The email included a link to our online survey hosted by Qualtrics (Provo, UT/Seattle, WA). We sent the invitations within three months of graduation, with two reminders after two and four weeks. Two institutions only approached their 2018 cohort as ethical clearance was not achieved in time for earlier participation. In total, 188 graduates with 30 different nationalities participated in our baseline survey, of whom 42 completed all follow-up questionnaires.

Third, we recruited interview candidates. After the first follow-up questionnaire (t1), we emailed all respondents (*n* = 47) inviting them to participate in online interviews to elaborate on their experiences. Nine graduates participated, of whom eight completed the full follow-up.

### Data collection

#### Survey

Our surveys were explorative, including multiple-choice and open-ended questions. We leveraged literature on international competencies, employability, and adaptation difficulties of IMGs to inform survey construction [9, 15, 21–23]. The baseline survey collected demographics, education information, as well as career preferences and experienced job preparedness. In the follow-up questionnaires, we asked about participants’ country of residence, employment status, experienced job requirements and their views on curriculum evaluation. Both baseline and follow-up surveys included questions around curriculum evaluation of the international medical program. We piloted the surveys with 25 graduates from two institutions who had graduated in 2016. This allowed for adjusting ambiguous questions and incorporating feedback on question clarity. Baseline and follow-up surveys are included in the Electronic Supplementary Material (ESM), parts A and B.

There were four data collection moments in total. We invited the t0 respondents for follow-up at six months after the baseline study (t1), then again a year later (t2) and two years later (t3, 2017 cohort only). Tab. 2 presents details on the data collection timeline and response rates.

**Table 2:** Timeline and response rate data collection

	Invitations	Responses T0 (graduation)	Responses T1	Responses T2	Responses T3
*Cohort 1 (graduation 2017; 5 institutions)*					
Timeline		April–September 2017	March–April 2018 (+ 6–12 months)	March–April 2019 (+ 1 year)	March–April 2020 (+ 1 year)
Responses	484	72	17	18	15
*Cohort 2 (graduation 2018; 7 institutions)*					
Timeline		April–September 2018	March–April 2019 (+ 6–12 months)	March–April 2020 (+ 1 year)	–
Responses	614	116	30	27	–
Total responses & response rate	1098	188 (17.1%)	47 (25%)	45 (23.9%)	15 (20.8%)

#### Interviews

We conducted a series of semi-structured interviews with nine participants to elaborate on the themes addressed in the survey and to get in-depth insight into individual experiences and their evolution. Interviews took place two (2018 cohort) or three times (2017 cohort) at one-year intervals, after the t1, t2 and t3 surveys. In total, 20 interviews took place with nine graduates. EB and TvR conducted the interviews online in English (*n* = 7) and Dutch (*n* = 2). Interviews were audio recorded and transcribed verbatim, lasting between 26 and 49 min. The interview guides are included in ESM C and D.

### Data analysis

Our mixed-method study had a sequential design and data analysis took place alongside the different data collection moments. We analysed the datasets separately and then used the qualitative data to explain and elaborate on the quantitative findings.

We performed summary statistics of the quantitative survey data in Excel 2010 to describe the main demographic data and survey responses.

For the qualitative data from the surveys’ open-ended questions and the interviews, we performed Template Analysis [24]. The initial template included a priori themes around study choice motivation, career preparedness and curriculum evaluation, drawn from the literature. Data analysis started after all t1 interviews took place and continued iteratively throughout the further study phases. EB and TvR independently coded three interviews first, then discussed codes and emerging themes and, in line with the Template Analysis method, modified the template accordingly before continuing analysis. The full research team met frequently during this process to reach agreement on data interpretation, additional themes and possible relationships between them until consensus was reached. We then applied the final template to the surveys’ open-ended questions and final interview round. We observed two distinct patterns when clustering the themes during our analysis of the interviews, which we described as two student profiles. We then reviewed the survey questions where participants elaborated on their study motivations and career ambitions to gauge the spread of these profiles across our cohorts. We obtained feedback from the interview participants on a summary of data interpretation as a member check, which led to clarification and nuances of comments in two cases.

### Research team & reflexivity

The research team is based at Maastricht University in the Netherlands, one of the participating institutions. All authors are primarily education researchers, with backgrounds in medicine (EB), public administration (TvR), social sciences (JF) and educational sciences (ED). Three of us (EB, JF, ED) also teach or have taught in the Maastricht International Medical Program, which not only inspired the study, but also shaped our assumptions and the research itself. We therefore valued the outsider’s perspective that TvR brought to the team. The first author (EB) kept a reflexivity journal throughout the study, and during data analysis we explicitly sought for and reported those findings that were not in line with our initial assumptions.

## Results

Our analysis of the career motivations and considerations that participants described, revealed two distinct profiles of students in IMPs, presented below as a ‘tale of two graduates’. Next, we present curriculum suggestions, based on the transition challenges, job requirements and curriculum experience that participants discussed. The analysis of the open-ended survey questions and the longitudinal interview series leads in the presentation of our results. Tab. 3 presents demographic data, information on the participants’ career choices and curriculum evaluation. Additional quantitative data is integrated in our presentation of the findings.

**Table 3:** Participant characteristics, career choice and location, curriculum evaluation and perception of international career at graduation and 2 years after graduation from an International Medical Program (IMP)

Participant characteristics	At T0 (*n* = 188) ^a^		At T2 (*n* = 45) ^a^	
Female	59%			
Mean age at T0	26			
**Residence location post-graduation**	**At T0**		**At T2**	
Returned to home country	41.7%		40.9%	
Stayed in country of their IMP	24.3%		2.3%	
Studied IMP in home country and stayed	26.2%		22.7%	
Moved to 3rd country	7.7%		31.8%	
*Unknown*	*–*		* 2.3%*	
**Career choice**	**At T0**		**At T2**	
Patient care	76.9%		78.7%	
Research	7.4%		4.2%	
Further degree study	8.3%		8.5%	
Other/don’t know	7.4%		4.2%	
**Specialty choice**	**At T0**		**At T2**	
Clinical sub-specialty	93.5%		97.3%	
Tropical medicine / International health	4.6%		2.7%	
Don’t know	1.9%		–	
	**At T0**	**At T1**	**At T2**	**At T3**
**On a scale from 1–10, “1” meaning not well at all and “10” meaning extremely well, how well do you think your international medical program has prepared you for your career?**	6.41	6.65	7.11	7.39
**International career**	**Do you envision yourself to have an international career? (T0)**		**Do you consider your current career to be international? (T2)**	
Yes	57.4%		61.4%	
No	8.3%		38.6%	
Maybe	34.3%		–	

^a^ Not all participants filled out all survey items. We report percentages of the group that filled out the item of interest

### Student profiles in IMPs

We identified two student profiles during our analysis, presented as the stories of ‘Anthony’, who pursued his medical studies ‘anyhow’, and ‘Isabel’, who was explicitly looking for international benefits. Among the 99 (57%) participants that elaborated on their study motivations and career ambitions, 46% fit Anthony’s profile, and 34% Isabel’s. The other 19% mentioned, for example, program duration or reputation as their main motivation. The stories reveal motivations for joining an IMP, career goals, and also highlight the main transition difficulties graduates experienced in their early careers.

#### Anthony Anyhow

*Anthony grew up in country X and always wanted to become a doctor. Unfortunately, the competition for medical school in X was high. Anthony refused to let this block his dream and started exploring alternatives. He learned about the concept of “International Medical Programs”. Anxious, but excited, he applied for an English program of good reputation in country Y because *“(t)he tuition fee was lower than other universities and the school didn’t require the applicant to perform an [entrance] test” [Survey participant #10, t0].* Studying medicine was hard work, and doing so in an unfamiliar country in his second language made it even more challenging. After six years of perseverance he graduated and achieved his goal of becoming a doctor*.

*Anthony moved back to his home country, hoping to find a junior doctor position. Getting his license was not as easy as expected, though. His foreign qualifications were not easily transferred and he had to take additional exams. Job applications were tough, as future employers were unconvinced of the quality of this unfamiliar international education. He felt his *“chances of getting into super competitive residencies afterwards [were] considerably lower” [Interview candidate #8, t1]* because of having studied in the IMP.*

*At last, he found a job in a hospital near his hometown. The first year came with many new challenges, as is true for any young doctor—however, Anthony felt disadvantaged because he had less clinical experience than his peers who had studied medicine in X and felt unfamiliar with the healthcare system*.

[The IMP] was a lot of theory, so I really got the theory part but yeah (…) We do meet patients but it’s not like, it’s not the same as when you work by yourself as a doctor. (…) I mean I can only compare myself to the doctors I have met now when I work and I feel like the doctors that are educated [locally], they have more practical skills. [Interview candidate #7, t1]

*He does not regret his choice though, and with the years his confidence has grown. Two years after graduation, he feels a part of the X healthcare system and having studied abroad feels like an adventure long ago, nice yet tough, that ultimately helped him achieve his dream*.

#### Isabel international benefits

*Isabel always dreamed of becoming a doctor. She grew up in country W, with a strong interest in travelling. She could picture herself practicing abroad. When learning about “International Medical Programs” she was immediately interested. This sounded like the ideal combination of medical education with studying in a foreign language and the additional benefit of meeting people from all over the world*.

*Isabel explored different IMP options in both her home country and abroad, and chose one in country Z, because she *“want[ed] to have a whole new experience and develop [her]self in a new environment” [Survey participant #182, t0].

*Studying medicine was hard work, and the student diversity indeed offered many options for intercultural learning. She graduated with many options in her mind. A career in a lower income country? Moving back to W? Or maybe staying in Z—as she had learned to appreciate the country, the language and the people*.

I always thought I would end up somewhere in South America (…) and then Tanzania happened so Africa kind of got me. And I’m now planning on visiting some more places. Could be anywhere actually. [Interview candidate #1, t1]

*Isabel felt the IMP was a strong asset on her CV. However, logistic challenges eventually limited her options. It was difficult to determine exactly what was needed to get a medical license in other countries, and it was costly and time consuming to prepare all exams and paperwork. Eventually, she moved back to W for specialty training, with all the additional experiences in her pocket. The first months were challenging, for sure, catching up with the local healthcare system and brushing up on her mother tongue*.

I was super worried in the beginning that (…) I would be lost and everything. But in the end, it didn’t [turn out that way], like the first weeks of course were hard, but they were super nice and helped me to learn. [Interview candidate #6, t2]

*A few years into her specialty program, Isabel speaks fondly of her IMP experience and, sometimes, still dreams about future practice abroad*.

### IMP curriculum suggestions

Analysing the participants’ experienced job requirements and their evaluation of the IMP curriculum’s success in preparing them for the job market, we identified two sets of curriculum suggestions for IMPs: specific international knowledge and skills, and guidance around international career preparation.

#### Knowledge & skills

Graduates across the institutions generally agreed that their curriculum had prepared them well in terms of medical knowledge and clinical skills and responded in an increasingly positive way over time to the question *“On a scale from 1–10, how well do you think your IMP has prepared you for your career?”* (Tab. 3). Interested in potential curriculum adaptations, we specifically looked into the reasons that respondents gave for a low mark to that question, and into the final survey item: *“Based on your current experiences, do you have any other suggestions to change the medical curriculum at your institute to better fit the requirements of international students and their future careers?”*

The most frequently discussed issues were clinical exposure and language. In some institutions, graduates felt they had little clinical experience overall, and many elaborated on the quality of clinical education during rotations heavily depending on patient interaction. Graduates agreed it was crucial to learn the local language in the pre-clinical years, as was common in some but not all of the IMPs in this study.

The education I received focused more on theory and not on practical knowledge. (…) Language barriers prevented me from gaining enough knowledge about how to communicate with the patient and be comfortable with my role as a doctor. I feel completely unprepared and I think will end up spending the first months of my career in fear. *[Survey participant #173, t0]*

We were particularly interested in international and intercultural competencies graduates needed in their early careers. However, these questions did not prompt elaborate responses in the survey nor interviews. Contrary to our expectations, a small minority (< 5%, see Tab. 3) considered and eventually pursued a career in global or international health. And while 61% considered their career to be international (at t2), this was largely explained as using international literature, and to a lesser extent as collaborating with foreign co-workers.

Graduates flagged our pre-defined international competencies such as global epidemiology, understanding of health systems and intercultural communication as ‘important’ to their current jobs. However, only few mentioned these topics in their suggestions for IMP curricula.

And then I would indeed mainly focus on, yes, expectations within different cultures. And what kind of position a doctor has in society. And also keep an eye on what are taboos in certain societies. *[Interview candidate #2, t1]*

Generally, when graduates mentioned lacking specific knowledge or skills compared to their peers, they acknowledged that no curriculum can cover all locally required knowledge of all potential destinations and accepted that it was their own responsibility to bridge these gaps.

#### International transition guidance

Almost half (49.4%) of respondents, like both Isabel and Anthony, crossed borders directly after graduation to either their home country or a new destination. Two years later, this number had increased to 73% and essentially only those native to the study country remained (Tab. 3). The migrating group reported specific challenges and suggested curriculum elements that would have helped them to smooth the international transitions. Concise suggestions were written in the surveys, and as five of our interview candidates also experienced this international transition, we further discussed potential curriculum interventions during the follow-up interviews.

*Reflecting on her final years in the IMP, Isabel mentioned that she had felt lost and left alone in finding out about her career options and preferences*.

All the information about this [cross-border] transfer I found by myself. There was only some information about the USMLE and about moving to the UK—but only informally because of the background of certain professors. [Interview candidate #11, t2]

*She would have liked her university to provide international career events, including how to find information on licensing examinations and degree registration. Isabel also suggested IMPs to make better use of their diverse alumni networks. This already happened informally, mainly through social media, but she thought that *“if the alumni connection [would be] strengthened not just by the student council body but also by the school itself, (…) that could be very fruitful” [Interview candidate #8, t2].* Isabel also suggested inviting international speakers from a broad range of countries and specialties to offer inspiration and insight in international careers*.

*Anthony always knew that he would move back to his home country. He thought the IMP could have better supported his preparation for remigration, for example, by allowing curricular time to prepare for his licensing exam. Ideally, the teaching staff would have been available to assist in this process*.

I have seen [IMPs] that help their students by building up their CVs with international courses (…) or even motivate and help their students to do the USMLE exams. In general, these colleges also prepare their students for what’s after their graduation internationally. [Survey participant #187, t0]

*Anthony also wished he’d had the opportunity to do placements in his home country. This would not only have helped him in career orientation, but also in networking to increase his success rate in job applications*.

The schools should probably ramp-up their effort and their support for people getting electives and to encourage it because if people are going to end up in [countries], they need to train there and have a chance to acclimate themselves before they even apply. [Interview candidate #8, t2]

In summary, graduates did not report a lack of specific academic content, but their experiences mainly point towards curriculum adaptation on support levels and international transition guidance.

## Discussion

Following IMP graduates from seven different universities into their early international careers, we identified two typical student profiles and a need for better international transition guidance.

The identification of these two profiles in part aligns with our earlier work around IMPs. In three different institutions, we studied curricula and interviewed teachers about their curriculum design experiences [25]. We found that staff perceived IMPs as either intending to deliver a ‘universal professional’ who could practice anywhere, or to prepare special ‘global physicians’ for international or global health career paths. Each of these perspectives was associated with specific curriculum design challenges. What did not emerge from that study, however, was a recognition that the IMP student population consists of both ‘Anthonys’ and ‘Isabels’. This finding adds an important insight and further complexity to IMP curriculum design as these profiles represent different interests. We recommend careful exploration of incoming students’ intentions and expectations to further inform curriculum design in practice as well as at a scholarly level.

Some challenges that our participants experienced during the transition into their early career match those of any new doctor shortly after graduation. For example, the experienced lack of clinical skills and sudden high responsibility are commonly reported [26, 27]. Furthermore, our findings on challenges specific for cross-border transitions align with research on International Medical Graduates, for example, regarding familiarization with health systems, administration, hierarchies and language [9, 10, 28]. However, where previous studies focused on remediation and interventions at the postgraduate level in the destination countries [29–31], our study adds the undergraduate perspective, where guidance, networking and entrance exam preparation could all facilitate cross-border transitions. We encourage further research on the design and effect of such pre-transition interventions.

Our study also contributes to our understanding of curriculum internationalization. Defined as “incorporating intercultural, international and global dimensions into higher education curricula”, this process aims to better prepare university graduates for a globalized, interconnected world [13]. Previous work has largely focused on incorporating these dimensions into the content of curricula, addressing, for example, global disease burden and immigrant health, cultural competence or mobility programs such as electives in low resource settings [32, 33]. Remarkably, the graduates in this study, who clearly operate in a globalized, interconnected world, hardly mentioned these themes when invited to share ideas for curriculum adaptations. We do learn from them that career guidance and international transition orientation are particularly valued and thus such support elements should not be neglected in program design, whether in IMPs or in other programs that wish to internationalize their curriculum.

This study is, to our knowledge, unique in the diversity of both institutions and participants, its mixed-methods approach and three-year longitudinal follow-up. The study limitations are inherent to this design: organizing recruitment through different local procedures was challenging with a relatively low response rate as a result. Also, as with many longitudinal studies, the loss to follow-up, especially between the first and second data collection point, was substantial. A self-selection bias, leading to a sample of participants, particularly among the interview candidates, who share a more positive perspective on IMPs, is plausible. We therefore purposely monitored the more critical and deviant voices throughout the follow-up and analysis. The authors are all from one of the participating institutions and therefore may have missed certain nuances of the other institutional contexts.

## Conclusion

IMP graduates generally regard their curriculum content effective in terms of career preparation. However, they miss support in practical aspects of international career orientation and preparation. Most IMPs essentially prepare their graduates for a career elsewhere. This entails a responsibility for cross-border transition guidance that is currently lacking.

Besides posing challenges to curriculum design, globalization offers a range of opportunities that medical schools with and without IMPs insufficiently embrace. Sharing international graduates’ stories, for example, through strong alumni networks, is only one approach. There is a world to win.

## Supplementary Information

Annex A—Baseline Questionnaire

Annex B—Follow-up Questionnaire

Annex C—Interview guide T1

Annex D—Interview guide T2&3
