# Supplementary material for: Early career experiences of international medical program graduates: An international, longitudinal, mixed-methods study
Source: Perspect Med Educ. 2022 Jul 26;11(5):258–65. doi: 10.1007/s40037-022-00721-z (PMC9582102; doi:10.1007/s40037-022-00721-z)
Supplement: Supplementary file 1 — Annex A—Baseline Questionnaire [file 40037_2022_721_MOESM1_ESM.docx]

| **Annex A – Baseline Questionnaire** | | |
| --- | --- | --- |
|  |  |  |
| **#** | **question** | **answer format** |
|  |  |  |
| **A - General information** | | |
| 1 | What is your gender? | Male/female |
| 2 | What is your year of birth? | Drop-down menu |
| 3 | What is your current country of residence? | drop-down menu; single answer |
| 4 | What is your country of origin? | drop-down menu; single answer |
| 5a | What is your country of citizenship? | drop-down menu; single answer |
| 5b | If you currently have multiple citizenships, please select: | drop-down menu; multiple answer; N/A as option |
| 6 | In which country did you obtain your secondary school degree? | Drop-down menu; single answer |
| 7 | In which institute will or did you obtain your medical degree? | MCQ with all participating institutes; & ‘other, please specify’ |
| 8a | Did you at any point switch or transfer to a different medical school during your medical studies? | Yes/No |
| 8b | If yes, in of after what year did you switch? | Year 1/2/3/4/5/6 |
| 8c | If yes, where did you start off your medical studies? | Open |
| 9 | What is your (expected) graduation date? | Month, Year |
| 10 | What was your most important argument to choose this programme at this institute for your medical studies? | Open-ended |
| 11 | What was the most important reason that you did not decide to study a standard medical programme in your home country? | Open-ended |
|  |  |  |
| **B – Career choice** | | |
| 12 | In what sector do you plan to work/study after graduation? | - patient care (incl residency training) - further degree study; other than medical specialty training (incl MSc programs) - research; including PhD training - governmental organization - non-governmental organization - higher education & teaching - private sector (including pharmaceutical companies; consultancy agencies) - self-employment, please specify - don’t know yet - other, please specify |
| 13 | In what field or medical discipline do you want to work or study after graduation? | - Internal medicine or subspecialties - Surgery or surgical subspecialties - Emergency medicine - Family medicine or primary health care - Obstetrics & Gynaecology - Paediatrics - Psychiatry - Public Health - Tropical Medicine or International Health - Other; please specify ….. |
| 14a | What is your preferred country for work or study after graduation? | drop-down menu; single answer |
| 14b | If applicable; in which other country or countries do you consider to apply for work or study after graduation? | drop-down menu; multiple answer; N/A as option |
| 15a | Do you envision yourself to have an international career? | Yes/no |
| 15b | If yes, please explain briefly how you imagine an international career | Open-ended |
|  |  |  |
| **C – Curriculum evaluation** | | |
| 16a | How well do you think your medical programme has prepared you for your career? | Scale 1-10 |
| 16b | Please explain briefly | Open ended |
| 17 | Based on your current experiences; how would you change the medical curriculum at your institute to better fit the requirements of international students and their future careers? | Open ended |
| 18 | Do you have any further remarks about this survey or about your experiences as a recent graduate from an internationalized medical programme? | Open ended |
