# Supplementary material for: Early career experiences of international medical program graduates: An international, longitudinal, mixed-methods study
Source: Perspect Med Educ. 2022 Jul 26;11(5):258–65. doi: 10.1007/s40037-022-00721-z (PMC9582102; doi:10.1007/s40037-022-00721-z)
Supplement: Supplementary file 2 — Annex B—Follow-up Questionnaire [file 40037_2022_721_MOESM2_ESM.docx]

|  | **Annex B – Follow-up Questionnaire** | |
| --- | --- | --- |
|  |  |  |
| ***#*** | ***question*** | ***answer format*** |
|  |  |  |
| **A - Career choice** | | |
| 1 | What is your current employment status? | - paid employment (including specialty training & PhD training) - self-employed - further degree study other than medical specialty training (incl MSc programs) - unemployed - other, please specify |
| 2 | In what country do you currently reside? | drop-down menu; single answer |
|  | *[IF ‘further degree study’ in Q1; answer Q3-5]* |  |
| 3 | What is the name of your study programme, institute & country? | Open-ended |
| 4 | What is your expected graduation date for this programme? | Month, year |
| 5 | Why did you choose to obtain this degree? | Open-ended |
|  | *[IF ‘paid employment’ or ‘self-employed’ in Q1; answer Q6-8]* |  |
| 6 | In what sector do currently work? | - patient care (incl residency training) - research; including PhD training - governmental organization - non-governmental organization - higher education & teaching - private sector (including pharmaceutical companies; consultancy agencies) - don’t know yet - other, please specify |
| 7 | In what field or medical discipline is your current position? | - Internal medicine or subspecialties - Surgery or surgical subspecialties - Emergency medicine - Family medicine or primary health care - Obstetrics & Gynaecology - Paediatrics - Psychiatry - Public Health - Tropical Medicine or International Health - Other; please specify ….. |
| 8a | Does your current job involve any   - international travel; - communication with colleagues from different nationalities; - communication with patients from different nationalities; - international themes in the content of your work? | “Rubric”:  0/1/2-3/>3 times per year daily/weekly/monthly/sometimes, but not every month/never  daily/weekly/monthly/sometimes, but not every month/never |
| 8b | If yes any of the above, please explain briefly the nature of the international elements that you encounter in your work | Open-ended |
|  | *[for all]* |  |
| 9 | Did you take any additional courses or training since graduation, including any that were mandatory or offered by your employer? | Yes/No |
| 10 | If yes, what course(s) did you take and why? | Open-ended |
|  |  |  |
| **B - Job requirements & Curriculum alignment** | | |
| 11a | How well do you think your medical programme has prepared you for your career? | Scale 1-10 |
| 11b | Please explain briefly | Open ended |
| 12a | Do you experience any specific benefits of having studied in an international programme? | Yes/No |
| 12b | Please explain briefly | Open-ended |
| 13a | Do you experience any disadvantages because of having studied in an international programme? | Yes/No |
| 13b | Please explain briefly | Open-ended |
| 14 | How challenging do you perceive adaptation to your current work situation in terms of   - Balancing professional and personal life - Social adjustment - Isolation from family - Communication with the interprofessional team - Communication with patients - Local language and slang - Local healthcare system - Local hospital system and structure - Using evidence-based medicine - Medical documentation - Specialty specific clinical knowledge and skills | 4 pt likert   - very challenging - somewhat challenging - somewhat easy - very easy   & * Not Applicable |
| 15a | How important is each of the following skills and abilities to your current job or study?   - Intercultural communication - Foreign language skills (other than your native language) - Global disease epidemiology (other than the epidemiology in the country where you trained) - Understanding of a health care system different than the one in the country where you trained - Understanding of immigrant health - Social determinants of health - International collaboration - Adapting diagnostic & therapeutic decisions to the level of (technological or financial) resource availability | 4 pt likert   - Not important - A little important - Important - Essential |
| 15b | Looking back to your medical degree program, did you receive training that helped you develop the following skills and abilities?  *Same list as above* | - effective training provided - training provided, but was NOT effective - no training, but training would have been helpful   * no training, and training is not necessary |
| 16 | Based on your current experiences; how would you change the medical curriculum at your institute to better fit the requirements of international students and their future careers? | - Open-ended |
| 17 | Do you have any further remarks about this survey or about your experiences as a recent graduate from an internationalized medical programme? | - Open-ended |
