# Supplementary material for: Early career experiences of international medical program graduates: An international, longitudinal, mixed-methods study
Source: Perspect Med Educ. 2022 Jul 26;11(5):258–65. doi: 10.1007/s40037-022-00721-z (PMC9582102; doi:10.1007/s40037-022-00721-z)
Supplement: Supplementary file 3 — Annex C—Interview guide T1 [file 40037_2022_721_MOESM3_ESM.docx]

**Annex C – Interview guide T1**

| *Main topics & questions* | *Probes / follow-up questions* |
| --- | --- |
| **Programme choice & evaluation** | |
| Why did you choose to study programme X? | Positive choice   - Travel/study abroad - International programme - Quality - Career perspective - Language   Negative choice   - Not selected - Quality at home - Not first choice - Financial restrictions   No choice (scholarship) |
| Did programme X fulfil your expectations? | Examples of surprisingly positive or negative experiences |
| **Career choice** | |
| What do you currently do and why did you choose it? | Would you have chosen differently after a ‘regular’ programme? |
| How well did programme X prepare you for your current job?  What aspect of your current job were you least prepared for? | Consider   - Medical expertise - Communication - Health systems - Cultural adaptation - Collaboration   Discuss examples of situations that he/she felt unprepared for.  Would that have been different after a ‘regular’ programme? |
| Do you envision yourself to have an international career? | What does that mean to you? Consider   - International patients - International colleagues - International travel - International ‘content’ |
| Does your current job require any ‘international skills’? | How well prepared do you feel for those? |
| **Curriculum alignment** | |
| Do you think there were any gaps in the international programme, things that you should have been taught to prepare well for your current position? | Or for your future career? Consider   - Medical expertise - Clinical skills/experience - International exam preparation - Global health topics - Travel/exchange opportunities   Discuss examples of situations where these gaps were apparent |
| Did you take any additional training since graduation? | What kind of training?  Would that be useful/necessary to include in undergraduate education? For regular/international students? |
| Do you experience any benefits of having studied in an international programme? | Could include   - Language skills - Degree value/reputation - Health system knowledge - Intercultural skills - Being open to others   Note examples of beneficial situations, eg   - Successful job application - Patient cases - Other work situations |
| And are there any disadvantages because of that? | Could include   - Preference for local graduates - Lack of knowledge/experience (health system; disease pattern; logistics) - Language barrier in studying limits learning - Racism & prejudice   Note examples of adverse events   - Discrimination - Patient cases - Hospital logistics |
| **Closure** | |
| Is there anything else you would like to share? |  |
| Would you be willing to take part in a follow-up  interview after the next questionnaire round? |  |
| Are you willing to check a summary of the interviews for accuracy/agreement/comments? |  |
