# Supplementary material for: Early career experiences of international medical program graduates: An international, longitudinal, mixed-methods study
Source: Perspect Med Educ. 2022 Jul 26;11(5):258–65. doi: 10.1007/s40037-022-00721-z (PMC9582102; doi:10.1007/s40037-022-00721-z)
Supplement: Supplementary file 4 — Annex D—Interview guide T2&3 [file 40037_2022_721_MOESM4_ESM.docx]

| *Main topics & questions* | *Probes / follow-up questions* |
| --- | --- |
| **Career choice** | |
| What do you currently do and where? | *If changed since previous interview:*  Why did you choose this job/study/…? |
| How well do you feel your IMP prepared you for | Consider |
| your current job requirements? | - Medical expertise |
|  | - Communication |
|  | - Health systems |
|  | - Cultural adaptation |
|  | - Collaboration |
| What aspect of your current job were you least prepared for? | *Discuss examples of situations that he/she felt unprepared for.* |
|  | Would that have been different after a ‘regular’ |
|  | programme? |
| Does your current job require any ‘international skills’? | How well prepared do you feel for those? Consider   - International patients - International colleagues - International travel - International ‘content’ |
| What are your plans for the future?  Have your career choices been influenced by having studied in an international programme? | Do you envision yourself to have an international career?  What does that mean to you? |
| **General reflection** |  |
| Looking back now, would you make the same choice for an international programme? | Which considerations would that choice have? Would you advice others to study in an international  programme? |
| **Curriculum alignment** | |
| Based on your recent work experience, do you | Or for your future career? |
| think there were any gaps in the international | Consider |
| programme, things that you should have been | - Medical expertise |
| taught to prepare well for your current position? | - Clinical skills/experience |
|  | - International exam preparation |
|  | - Global health topics |
|  | - Travel/exchange opportunities |
|  | *Discuss examples of situations where these gaps were apparent* |
| Do you have any suggestions for additional curriculum content in international medical programmes (compared to ‘regular’ programmes)? | Consider   - Medical expertise - Clinical skills/experience - International exam preparation - Global health topics - Travel/exchange opportunities |
| Did you, since the previous interview, experience any benefits of having studied in an international  programme? | Could include   - Language skills - Degree value/reputation |

**Annex D – Interview guide T2&3**

|  | - Health system knowledge - Intercultural skills - Being open to others   Note examples of beneficial situations, eg   - Successful job application - Patient cases - Other work situations |
| --- | --- |
| And were there any disadvantages because of that? | Could include   - Preference for local graduates - Lack of knowledge/experience (health system; disease pattern; logistics) - Language barrier in studying limits learning - Racism & prejudice   Note examples of adverse events   - Discrimination - Patient cases - Hospital logistics |
| **Closure** | |
| Is there anything else you would like to share? |  |
| Are you willing to check a summary of the  interviews for accuracy/agreement/comments? |  |
